# A Novel Way for Efficient and Safe Posterior Fossa Relaxation: The Extradural Opening of the Cisterna Magna in Retrosigmoid Craniotomy

**DOI:** 10.7759/cureus.67841

**Published:** 2024-08-26

**Authors:** Ziya Hamelinck, Dieter Thijs, Bart Feyen, Maarten Vanloon, Vincent Van Rompaey, Tomas Menovsky

**Affiliations:** 1 Department of Neurosurgery, Antwerp University Hospital, Antwerp, BEL; 2 Faculty of Health, Medicine and Life Sciences, Maastricht University, Maastricht, NLD; 3 Department of Otorhinolaryngology and Head and Neck Oncology, Antwerp University Hospital, Antwerp, BEL

**Keywords:** posterior fossa, extradural, surgical technique, cisterna magna, cerebrospinal fluid, retrosigmoid craniotomy

## Abstract

Cerebrospinal fluid drainage is a common practice to provide brain relaxation during intradural surgery. In retrosigmoid approaches, cerebrospinal fluid can be drained from the cisterna magna to provide brain relaxation in the posterior fossa. To our knowledge, most techniques to achieve cerebrospinal fluid release concern intradural opening of the cisterns. We describe a novel way for the extradural opening of the cisterna magna in retrosigmoid surgery that avoids direct cerebellar contact. Patients elected for surgical treatment of cerebellopontine angle tumors are positioned supine with a roll under the ipsilateral shoulder and the head turned to the contralateral side. After performing a retrosigmoid craniotomy, the surgical microscope is tilted, and the foramen magnum is approached extradurally. A horizontal dural slit is made at the level of the cisterna magna, and cerebrospinal fluid is drained without having direct cerebellar contact. After brain relaxation, the intradural surgery can proceed as usual. This slight adaptation for a very common practice avoids the need for direct cerebellar retraction when approaching the cisterna magna to drain cerebrospinal fluid. It is a clean and easy step to perform, that we believe improves surgical efficacy and could potentially diminish cerebellar harm because it obviates the need for intradural opening of the cisterna magna.

## Introduction

Cerebrospinal fluid (CSF) drainage is a common practice to provide brain relaxation during intradural surgery. This can be obtained with external ventricular drainage, lumbar drainage or opening of the arachnoid cisterns during surgery. In retrosigmoid (RS) approaches, CSF can be drained from the cisterna magna (CM) to provide brain relaxation in the posterior fossa. For a useful overview of the RS approach variations and CSF drainage options, we like to refer to the excellent paper of Basma et al. [1]. To our knowledge, most techniques to achieve CSF release from the cisterns concern an intradural approach and dissection. This usually necessitates cerebellar retraction and can result in cerebellar contusions and/or (micro)hemorrhages on the cerebellar surface, especially during surgery for large lesions. Majid et al. describe a suboccipital (retrosigmoid) approach with the patient positioned semi-sitting, where CSF is drained from the lateral cerebellomedullary cistern using a laterally convexed dural incision [2,3]. However, we describe a novel way for the extradural opening of the CM in RS surgery that avoids direct cerebellar contact.

## Technical report

Patients elected for surgical treatment of cerebellopontine angle tumors are positioned supine with a roll under the ipsilateral shoulder to rotate the sagittal axis of the head near parallel to the floor, the head elevated above the level of the heart and the neck slightly flexed. All patients consented to the retrosigmoid surgery and possible risks involved. After performing an RS craniotomy with a trepanation window of 3.5 to 4.5 cm, and before opening the dura, the microscope is tilted to provide a craniocaudal viewing angle towards the foramen magnum. With the suction device and a dissector, the dura is peeled off the posterior fossa skull base until the reflection point of the foramen magnum dura is encountered. The suction is kept in place to retract the cerebellar dura and a horizontal dural slit is made at the level of the cisterna magna with a number 11 blade. Usually, the arachnoid remains intact after the durotomy. Under high magnification, the dura and the arachnoid can be discerned from cerebellar tissue. The arachnoid membrane is then opened (e.g. with microscissors) after which ample CSF can be aspirated until sufficient relaxation of the cerebellar dura is obtained. In some patients, the arachnoid may protrude through the dural opening like an aneurysm which makes opening the arachnoid easier.

**Figure 1:**
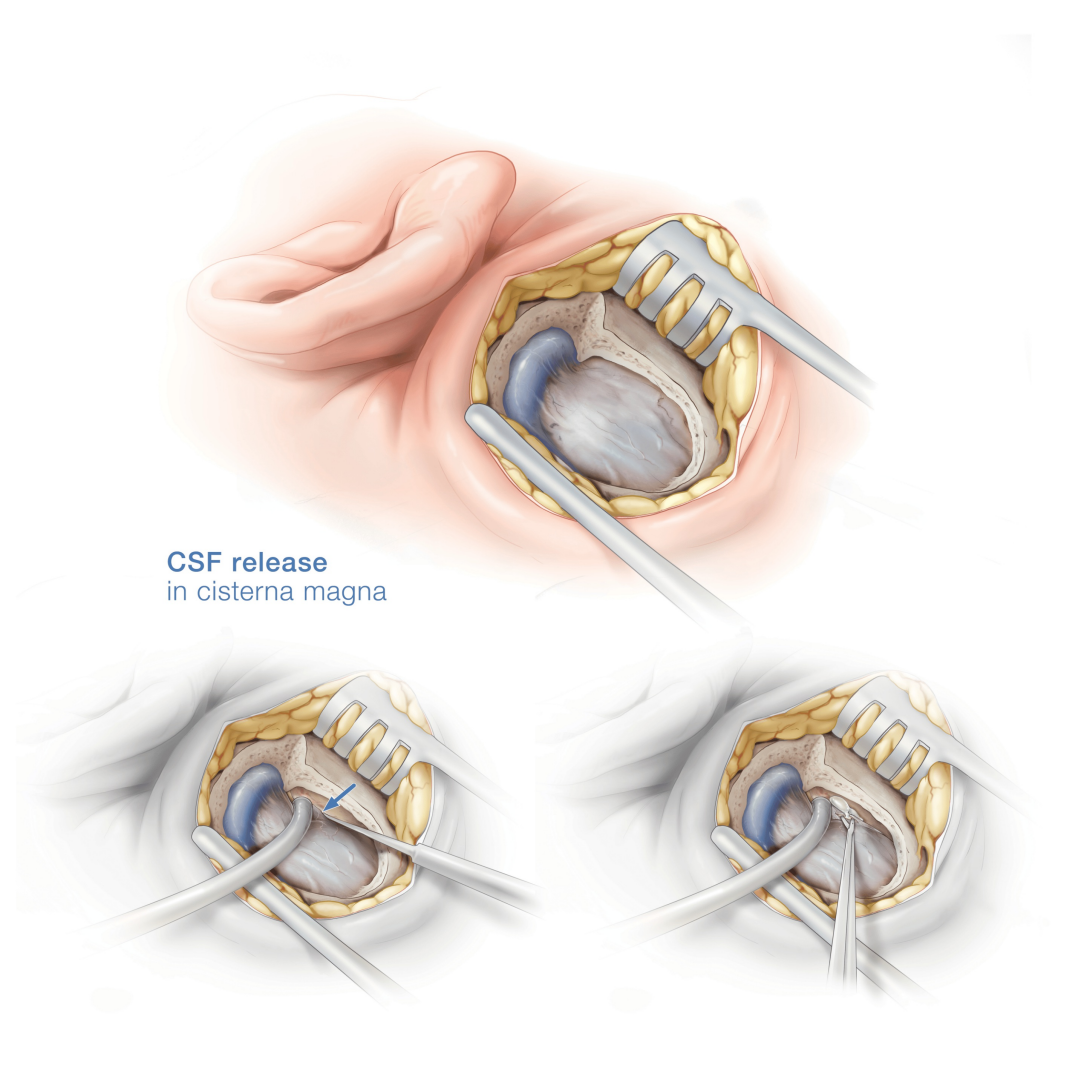
Original illustration of a right retrosigmoid craniotomy. The sigmoid sinus and retrosigmoid dura are exposed. With a dissector and the suction device, the dura is peeled away up to the bony edge of the foramen magnum. The dura is incised at the edge of the foramen, the arachnoid is opened with microscissors, and cerebrospinal fluid (CSF) is released.

After the posterior fossa relaxation, the dura is opened and the intradural surgery can start. At the end of the procedure the dural slit is sealed with a fibrinogen and thrombin matrix sponge (Tachosil, Takeda Austria GmbH, Linz, Austria) to prevent CSF leakage (Video 1). The technique and steps are summarized in Figure 1.

**Video 1:** Extradural opening of the cisterna magna.

Postoperative care, complication management and patient follow-up with this technique do not differ from the standard of care. In total, 25 patients were operated using this technique without intra- or postoperative complications. There were no patients with CSF hypotension syndrome postoperatively or during follow-up. The median follow-up was 2.5 years with a maximum follow-up duration of five years. The diameter of the lesions operated using this technique ranged from 3 to 4.5 cm.

## Discussion

Cisternal CSF release is a basic neurosurgical skill and of paramount importance to achieve brain relaxation during surgery. The standard, intradural approach to the CM, the cerebellomedullary or cerebellopontine cistern(s) necessitates cerebellar retraction and careful arachnoid dissection of the relevant cranial nerves before the cistern can be opened. Especially during surgery for large lesions, exerting mass effect in the posterior fossa, this carries a risk of cerebellar contusion and pial violation. A study by Huang et al. reported 250 cases (21.4%) suffering from disequilibrium after vestibular schwannoma resections through an RS approach [4]. The observed high incidence of disequilibrium in this study is hypothesized to be linked to excessive cerebellar stretching. With CSF release through the CM, excessive stretching of the cerebellum can be avoided. Furthermore, the authors suggest that bleeding from bridging veins and certain small arteries, including branches of the anterior inferior cerebellar artery (AICA) and the labyrinthine artery, can be prevented by moderating the decompression rate. Whether through cerebrospinal fluid release in the CM or tumor removal, it is recommended to avoid excessively rapid decompression [4]. Our technique, leaving most of the dura mater intact to provide protection of the cerebellar foliae, obviates the need for early arachnoid dissection of the lower cranial nerves and minimizes the risk of cerebellar contusion.

If venous bleeding is encountered during the durotomy, presumably from a dural venous channel, it can be easily controlled with irrigation and application of gelatin-based hemostat. The lower cranial nerves are in proximity to the dural slit. The suction device should be kept outside the dural slit and never advanced blindly through the durotomy. We have not encountered new lower cranial nerve deficits while performing this maneuver, as well as postoperatively and during follow-up. The whole extradural CSF release is performed using the surgical microscope with a magnification of 20. Postoperative T2-weighted MRI can illustrate the site of the durotomy due to small remnants of blood This could be relevant in patients with CSF hypotension syndrome to identify a source of leakage. However, in our population, we have not seen patients with CSF leakage or symptomatology. This is probably due to the small size of the durotomy, compared to other techniques, and the application of Tachosil during closure.

## Conclusions

In conclusion, the extradural opening of the cisterna magna is a new take on a classic technique. Simply stated, this adaptation is performing a classic cisterna magna approach but without the necessity of arachnoid dissection and leaving the protective dura over the cerebellum. The extradural opening of the cisterna magna in retrosigmoid surgery is an effective and safe way to achieve posterior fossa relaxation.
